# Cell engraftment, vascularization, and inflammation after treatment of equine distal limb wounds with endothelial colony forming cells encapsulated within hydrogel microspheres

**DOI:** 10.1186/s12917-020-2269-y

**Published:** 2020-02-04

**Authors:** Randolph L. Winter, Yuan Tian, Fred J. Caldwell, Wen J. Seeto, Jey W. Koehler, David A. Pascoe, Shirley Fan, Phillippe Gaillard, Elizabeth A. Lipke, Anne A. Wooldridge

**Affiliations:** 1 Department of Clinical Sciences, Auburn University, Auburn, AL USA; 2 Department of Clinical Sciences, Ohio State University, Columbus, OH USA; 3 Department of Chemical Engineering, Auburn University, Auburn, AL USA; 4 Department of Pathobiology, Auburn University, Auburn, AL USA; 5 School of Kinesiology, Auburn University, Auburn, AL USA; 6 Department of Mathematics, Auburn University, Auburn, AL USA

**Keywords:** Equine, Endothelial colony forming cells, Stem cell therapy, Regenerative medicine, Biomaterial, Microsphere, Skin, Wound

## Abstract

**Background:**

Endothelial colony forming cells (ECFCs) may be useful therapeutically in conditions with poor blood supply, such as distal limb wounds in the horse. Encapsulation of ECFCs into injectable hydrogel microspheres may ensure cell survival and cell localization to improve neovascularization and healing. Autologous ECFCs were isolated from 6 horses, labeled with quantum nanodots (QD), and a subset were encapsulated in poly(ethylene) glycol fibrinogen microspheres (PEG-Fb MS). Full-thickness dermal wounds were created on each distal limb and injected with empty PEG-Fb MS, serum, ECFCs, or ECFCs encapsulated into PEG- Fb MS (ECFC/MS). Analysis included wound surface area (WSA), granulation tissue scoring (GS), thermography, collagen density staining, and immunohistochemical staining for endothelial and inflammatory cells. The purpose of this study was to track cell location and evaluate wound vascularization and inflammatory response after injection of ECFC/MS or naked ECFCs in equine distal limb wounds.

**Results:**

ECFCs were found near and within newly formed blood vessels up to 3 weeks after injection. ECFC and ECFC/MS groups had the greatest blood vessel quantity at week 1 in the wound periphery. Wounds treated with ECFCs and ECFC/MS had the lowest density of neutrophils and macrophages at week 4. There were no significant effects of ECFC or ECFC/MS treatment on other measured parameters.

**Conclusions:**

Injection of microsphere encapsulated ECFCs was practical for clinical use and well-tolerated. The positive ECFC treatment effects on blood vessel density and wound inflammation warrant further investigation.

## Background

Poor or delayed healing of equine distal limb wounds is one of the most common problems faced by equine practitioners and horse owners. Distal limb wounds in the horse may have physical disruption of blood supply, excessive inflammation, and local ischemia and hypoxia, all of which contribute to the formation of exuberant granulation tissue (EGT) and slow wound healing times [1–4]. Distal limb wounds have protracted inflammation and slower epithelialization compared to body wounds, in part due to poor wound oxygenation [1–4]. Wounds with EGT have an abundant vascular supply [2, 4]; however, these vessels may be dysfunctional and occluded, thus promoting fibroproliferation [3]. Based on their ability to heal damaged blood vessels and form new vessels in vivo, endothelial colony forming cells (ECFCs) may be an attractive option for promoting wound healing in distal limb wounds of the horse.

Endothelial colony forming cells (ECFCs) have the ability to differentiate into mature endothelial cells and contribute to the formation of new blood vessels either directly or indirectly [5, 6]. ECFCs are considered ideal for in vivo clinical therapy for ischemic disease or vascular damage [7–10]. Although promising for use as an injectable treatment, there are important barriers to consider for ECFCs and other cellular therapies for tissue regeneration and repair. Direct injection of ECFCs or other cells into the wound or wound periphery is the most straightforward way to deliver these cells. However, the mechanical shear stress encountered during the injection through the needle is often enough to damage or kill many of the injected cells [11]. Additionally, cells that are injected into or around damaged areas such as inflamed wounds or infarcted areas experience a harsh environment with reactive oxygen species or damaging inflammatory cytokines [12]. The low retention rate of injected cells of ~ 5–9% in animal models of disease may be caused by cell death associated with either the injection or the harsh environment into which they are injected [13, 14].

Biomaterial scaffolds have a key role in stem cell therapy by ensuring that cells remain at the site of administration and by regulation of the stem cell microenvironment, which are essential to cell survival and engraftment [15]. To improve the reported 5–9% retention rate of injected cells [13, 14], evaluation of a combination of cells with a hydrogel scaffold was a goal of this study. Hydrogels are 3-dimensional scaffolds composed of hydrophilic polymer chains and can be cross-linked into any desired shape [16]. They can be coupled with oligopeptides or proteins to more strongly mimic extracellular matrix [16, 17]. Cells that are encapsulated in biomimetic hydrogels experience a microenvironment similar to the native extracellular environment [18]. This may in part explain why stem or progenitor cells that are encapsulated into hydrogels demonstrate proliferation and long-term survival [19]. Poly(ethylene) glycol coupled with fibrinogen (PEG-Fb) is a hybrid hydrogel coupled to a protein that has been utilized in cell therapy and enhances immediate survival of injected cells [11, 20]. Our group can consistently create cell laden PEG-Fb microspheres (MS) for injection [21]. The equine ECFC viability post-encapsulation was 97%, and cell marker expression and function was normal [21]. Encapsulated cells survived after injection through 18–23 gauge needles, and were detected in equine skin 1 week after injection in a previous study [21].

The purpose of this study was to track cell location and evaluate wound vascularization and inflammatory response after injection of PEG-Fb microsphere encapsulated or naked ECFCs in equine distal limb wounds and show proof of concept for clinical use of PEG-fb microsphere encapsulated ECFCs for regenerative therapy. We hypothesized that vascularization would be enhanced in wounds where ECFCs are injected and this effect would be augmented with PEG-Fb biomaterial encapsulation.

## Results

### Clinical findings

**Fig. 1:**
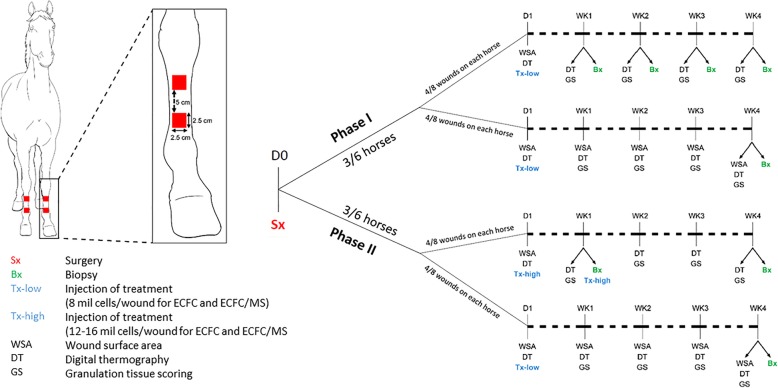
Schematic of study design. Two wounds were created on each metarcarpus/metatarsus. Tissues from biopsies were formalin fixed and paraffin embedded for immunohistochemistry or frozen in optimum cutting temperature media and imaged with confocal microscopy for cell tracking

**Fig. 2:**
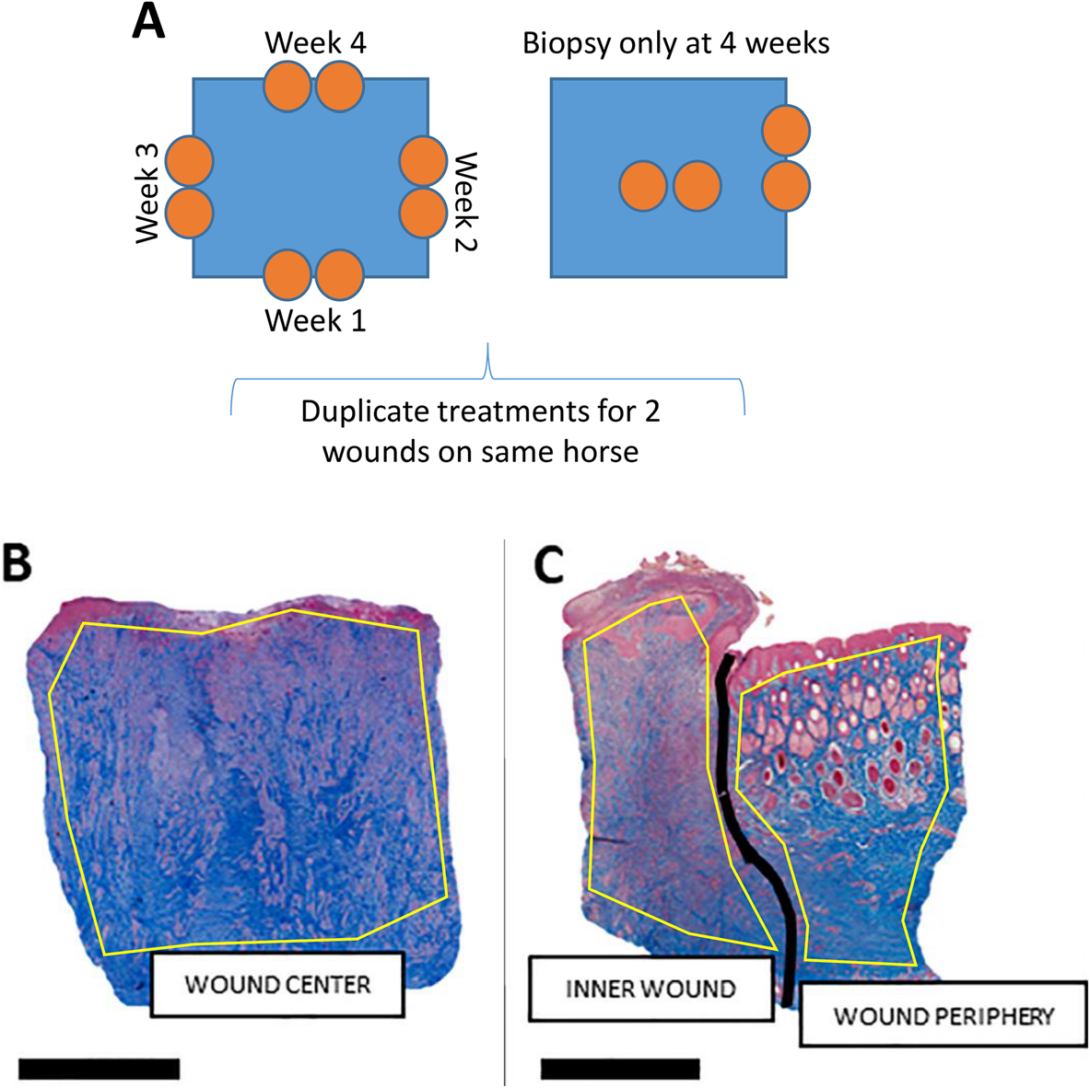
Schematic of wound biopsies and regions analyzed for quantification of immunohistochemical staining. (**a**) 2 wounds (blue squares) per horse were treated identically, but biopsied (orange circles) differently. 2 punch biopsies (one punch biopsy was fixed in formalin and the other was frozen) were collected at the leading edge of the wound once weekly, at the leading edge once 4 weeks after wound creation, or at the center of the wound once 4 weeks after wound creation. (**b,c**) Punch biopsies from wounds collected at either the center of the wound or the leading edge of showing the regions of interest (outlined in yellow) analyzed with the quantification software program. Biopsies are stained for collagen (blue) with Masson’s trichrome. “Inner Wound” (non-healed wound within advancing line of epithelialization); “Wound Periphery” (skin with epithelial cell cover, just on the periphery of the advancing line of epithelialization); and “Wound Center” (area at the center of the non-healed wound)

Six, healthy university-owned, adult horses (ages 9–26 years, 5 geldings, 1 mare) were utilized for autologous ECFC isolation and to create the distal limb wound model. Two, full thickness, 2.5 cm X 2.5 cm (6.25 cm^2^) wounds were created on each metacarpus and metatarsus, and each horse had 2 wounds created per limb (fore and hind) for a total of 8 wounds per horse (Fig. 1). Wounds were treated in duplicate with one of 4 treatments (ECFCs alone, ECFCs encapsulated in PEG-Fb MS, MS alone, or serum only). Biopsies were taken from the center and leading edge of the healing wound to evaluate specific regions histologically and to track injected cells (Fig. 2). The surgical wound model and treatments were generally well tolerated. One horse developed a fever after the surgery and initial treatments, but this was responsive to a single dose of flunixin meglumine (1 mg/kg IV). The same horse became febrile and developed swelling and stiffness in all 4 limbs after the first biopsy and was treated with one dose of flunixin meglumine (1 mg/kg IV) and a 10-day course of trimethoprim sulfamethoxazole (24 mg/kg PO). The swelling was not specific to any wound or treatment and completely resolved. There were statistically significant effects of individual horse (i.e. certain individuals had more complete wound healing, with less granulation tissue, faster than other individual horses regardless of other factors) and whether the wound was on a forelimb or a hindlimb (i.e. wounds on hind limbs had slower healing, with more granulation tissue regardless of other factors compared to forelimbs). These factors were accounted for in subsequent analyses, so that any significant differences found due to treatment group occurred despite these sources of variation.

### Changes in wound size over time

**Fig. 3:**
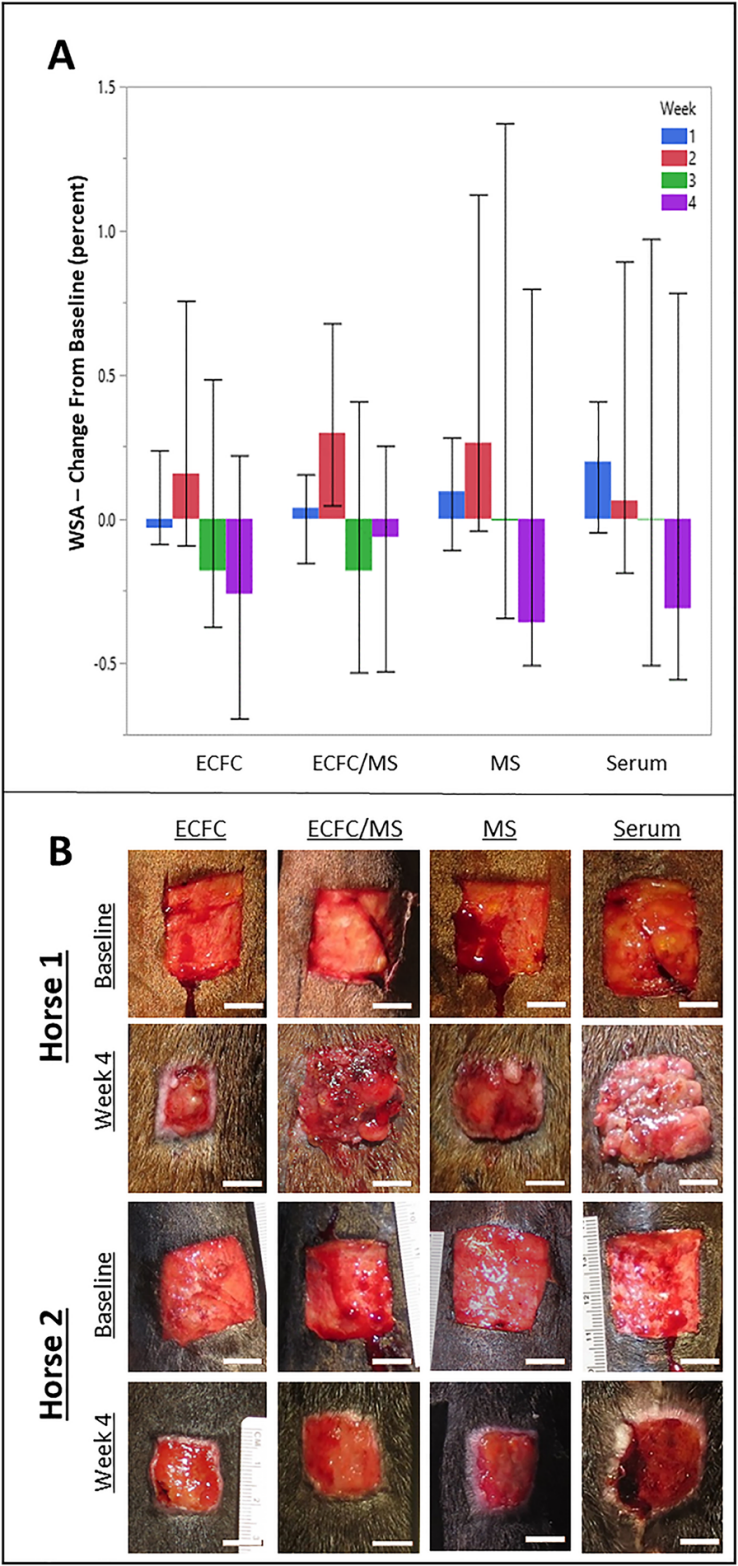
(**a**) Wound surface area (WSA) displayed as median and range for percent change from baseline wound for all treatment groups by week. (**b**) Photographs from 2 horses of wounds at baseline (24 h after wound creation) and at 4 weeks for all treatment groups. The variation between horses and different responses to treatments are apparent. ECFC and ECFC/MS treatment groups had 8 mil cells/wound injected at baseline. Scale bars are 1 cm

Comparisons in wound surface area (WSA) (Fig. 3) between treatments was only performed for wounds that were injected at 1 time point (LOW) and only biopsied at 4 weeks (24 wounds from 6 horses, 6 wounds per treatment). Treatment group did not significantly impact WSA change over time (Fig. 3). The WSA initially increased and then decreased over the course of the study in all wounds, and all horses were observed until all wounds were completely epithelialized (approximately 8 weeks). Wounds treated with ECFCs and ECFC/MS had the smallest WSA at week 3 (Fig. 3), but this was not significant (*P* = 0.767). WSA decreased in all wounds over time (*P* ≤ 0.001).

### Granulation tissue assessment

**Fig. 4:**
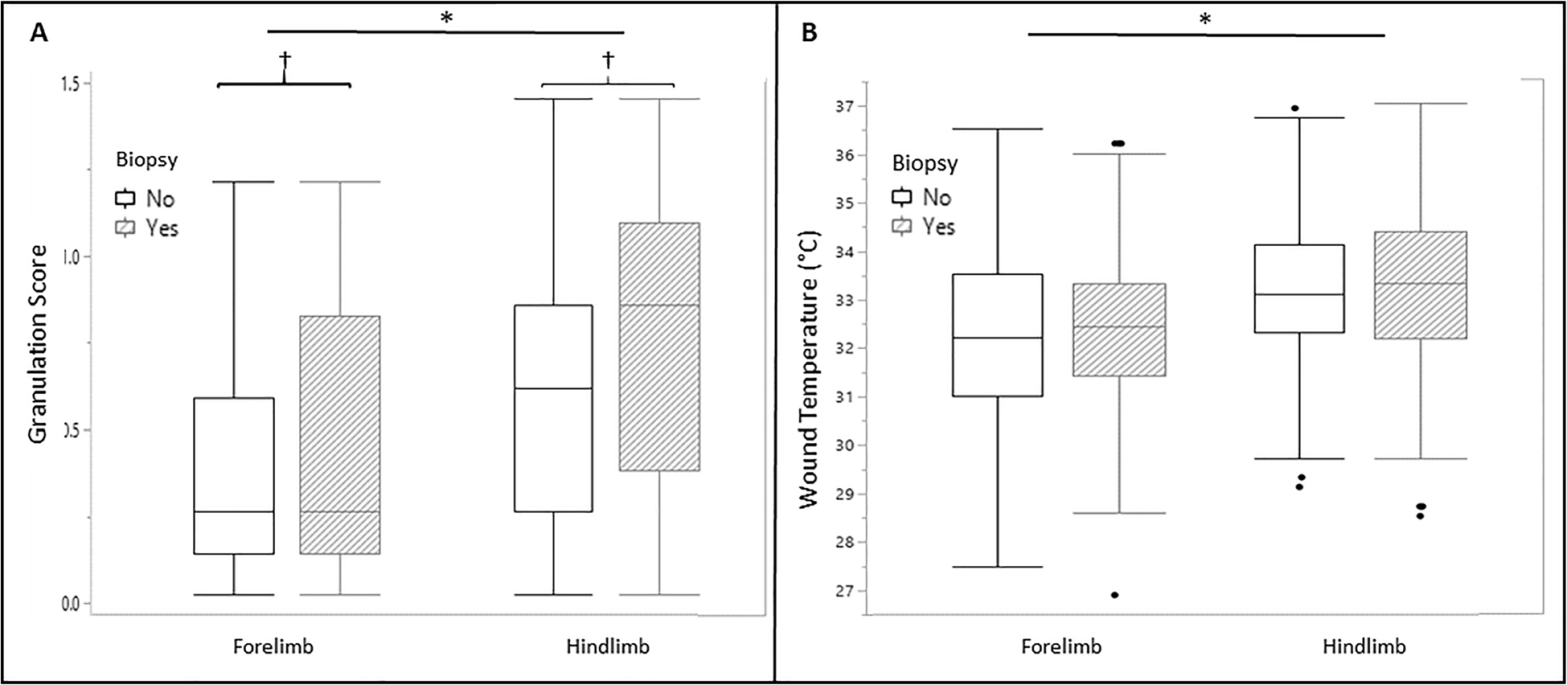
(**a**) Weighted granulation score for all wounds by whether the wound was on a forelimb or hind limb and whether the wound was biopsied. Data are presented as box-and-whisker plots, with median values represented by the horizontal lines and the interquartile range represented by the box. Black dots are values that are 1.5 times the box length above the 75th percentile. The ends of the whiskers represent the smallest and largest values not classified as outliers. (**b**) Wound temperature in °C for all wounds by whether the wound was on a forelimb or hind limb and whether the wound was biopsied. * signifies a significant difference in wound temperature and weighted granulation score between forelimbs and hindlimbs (*P* = < 0.05). † signifies a significant difference in weighted granulation score based on whether the wound was biopsied (*P* = < 0.05). Note that wounds on hindlimbs had higher temperatures and more granulation tissue compared to wounds on forelimb wounds, but taking biopsies only affected the severity of granulation tissue

Comparisons in granulation scores (GS) between treatments were performed for all wounds at weeks 1, 2, 3 and 4 (48 wounds from 6 horses, 12 per treatment) (Fig. 4). The effects of ECFC dose (HIGH vs LOW cell numbers) and injection frequency (once vs twice during the study) were not significant, and therefore only treatment group was included in the analysis. There was no effect of treatment on GS at any time point (*P* = 0.31). The GS were highest at week 3 (0.875, range 0.125–1.5) and lowest at week 1 (0.25, 0.0–1.0, *P ≤* 0.001). Hind limbs had higher GS than forelimbs (*P ≤* 0.001) (Fig. 4), with hind limbs having an Odds Ratio (OR) of 3.61 (95% CI 1.87–7.00) of having a weighted GS greater than 0.75. Wounds which were biopsied weekly also had higher scores than wounds biopsied only at week 4 (*P* = 0.023), with weekly biopsied wounds having an OR of 2.11 (95% CI 1.12–3.96) of having a weighted GS greater than 0.75.

### Thermographic assessment of wounds

Comparisons of thermography data between treatments were performed for all wounds at weeks 1, 2, 3 and 4 (48 wounds from 6 horses, 12 per treatment)(Fig. 4). The effects of ECFC dose (HIGH vs LOW cell numbers) and injection frequency (1 time vs 2 times during the study) were not significant. There was no effect on the % change in temperature due to treatment. Wound temperature % change acutely at week 1 was greatest for ECFC (+ 2.4%, − 7.19 to + 47.29%) and ECFC/MS (+ 3.23%, − 19.2 to + 12.03%) compared to MS alone (+ 2.14%, − 12.25 to + 15.11%) and serum (− 2.11%, − 9.76 to + 13.64%), but the effect of treatment group was not significant (*P* = 0.416). The effect of treatment group on wound temperature % change at week 4 was also not significant (*P* = 0.061). Factors that significantly affected wound temperature were time post injection (greatest temperature at week 1 and lowest at week 4, *P ≤* 0.001), location of measurement (measurements either 1 cm above or 1 cm below the wound were lower in temperature; *P* ≤ 0.001 and *P* = 0.004, respectfully), and limb (fore limbs were lower than hind limb wound temperatures (*P* = 0.01). There were no differences in wound temperature between proximal and distal wounds. Wounds that were biopsied did not have differences in wound temperature compared to wounds that were not biopsied (*P* = 0.368) (Fig. 4).

### Evaluation of collagen density

Comparisons of collagen density (quantified by Masson’s trichrome staining of wound biopsies) between treatments for individual regions (Inner Wound, Wound Periphery, Wound Center) were performed on all wounds biopsied at week 1 and week 4 (48 wounds from 6 horses, 12 per treatment) with different wound regions analyzed separately. Both serum and MS only treated wounds had small, but statistically significant changes in collagen density at week 1, but differences did not persist at week 4. Wounds injected with serum had increased collagen density in the Inner Wound at week 1 (*P* = 0.026), and those injected with MS alone had decreased collagen density in the Wound Periphery at week 1 (*P* = 0.037). None of the cell-based treatments had significant effects with any location or treatment.

### Assessment of vascularization

**Fig. 5:**
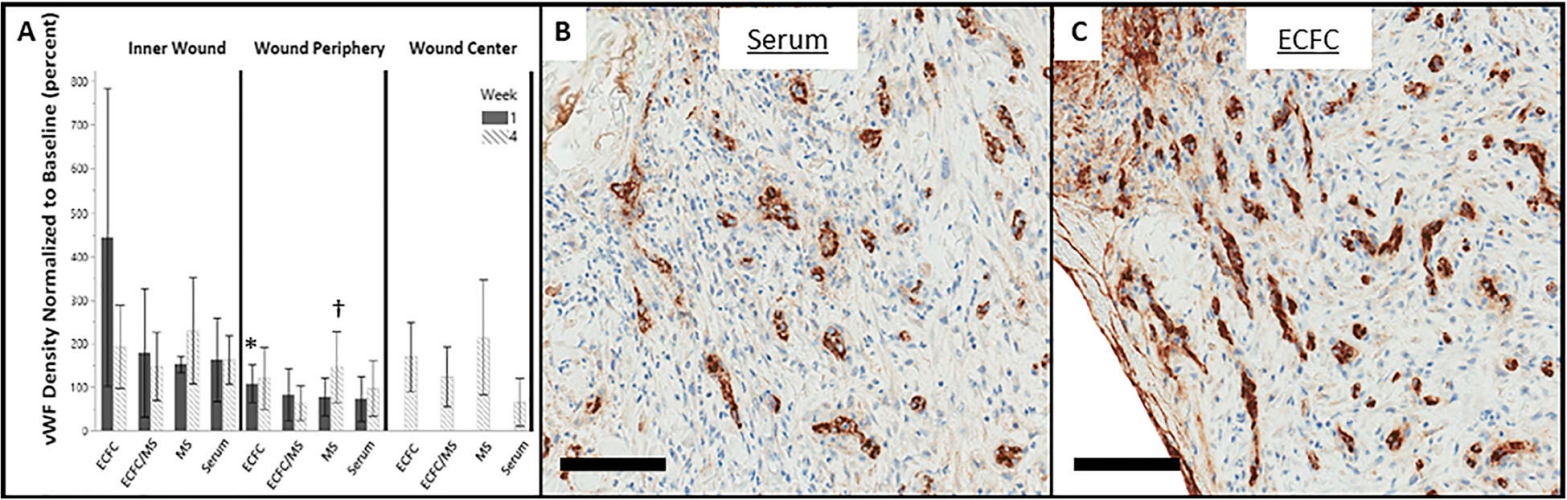
(**a**) Density of von Willebrand factor (vWF) staining (vascularization) normalized to baseline values at weeks 1 and 4 by treatment group in the Inner Wound, Wound Periphery, and Wound Center regions. Data are presented as means and standard deviation. The Wound Center region was only biopsied at week 4. (**b**) Representative photomicrograph of blood vessel density (brown stain) in the serum treatment group at the Inner Wound region at week 1. (**c**) Representative photomicrograph of blood vessel density in the ECFC treatment group at the Inner Wound region at week 1. Note the relative increase in blood vessel density in the ECFC treatment group compared to the Serum treatment group in (**b**) and (**c**). * indicates a significant difference in vascularization in the Wound Periphery region at week 1, with wounds treated with ECFC having greater blood vessel density compared to wounds treated with MS alone (*P* = < 0.05). † indicates a significant difference in vascularization in the Wound Periphery region at week 4, with wounds treated with MS alone having greater blood vessel density compared to all other treatment groups (*P* = < 0.05). Scale bars are 100 μm

Comparisons of blood vessel density (quantified by von Willebrand factor (vWF) staining of wound biopsies) (Fig. 5) between treatments were performed on all wounds biopsied at week 1 and week 4 (48 wounds from 6 horses, 12 wounds per treatment), and each region of the biopsy (Inner Wound, Wound Periphery, or Wound Center) was analyzed separately. Blood vessel density was greatest at week 1 in wounds treated with ECFCs or ECFC/MS (Fig. 5). The Inner Wound region at week 1 had the greatest density of blood vessels in wounds treated with ECFC and ECFC/MS, but was not significant (*P* = 0.173). The Wound Periphery regions at week 1 had overall differences in vascularization (*P* = 0.009); wounds treated with ECFCs and ECFC/MS also had the greatest density of blood vessels in the Wound Periphery, with wounds treated with ECFCs having a significantly greater density of blood vessels compared to wounds treated with MS alone (*P* = < 0.001) (Fig. 5). No significant differences in vascularization were observed in Wound Center regions (*P* = 0.157) or in the Inner Wound regions (*P* = 0.157) at week 4. In the Wound Periphery regions at week 4, there were significant differences in vascularization due to treatment group (*P* = 0.042), with the MS alone group having the greatest density of blood vessels.

### Assessment of inflammation

**Fig. 6:**
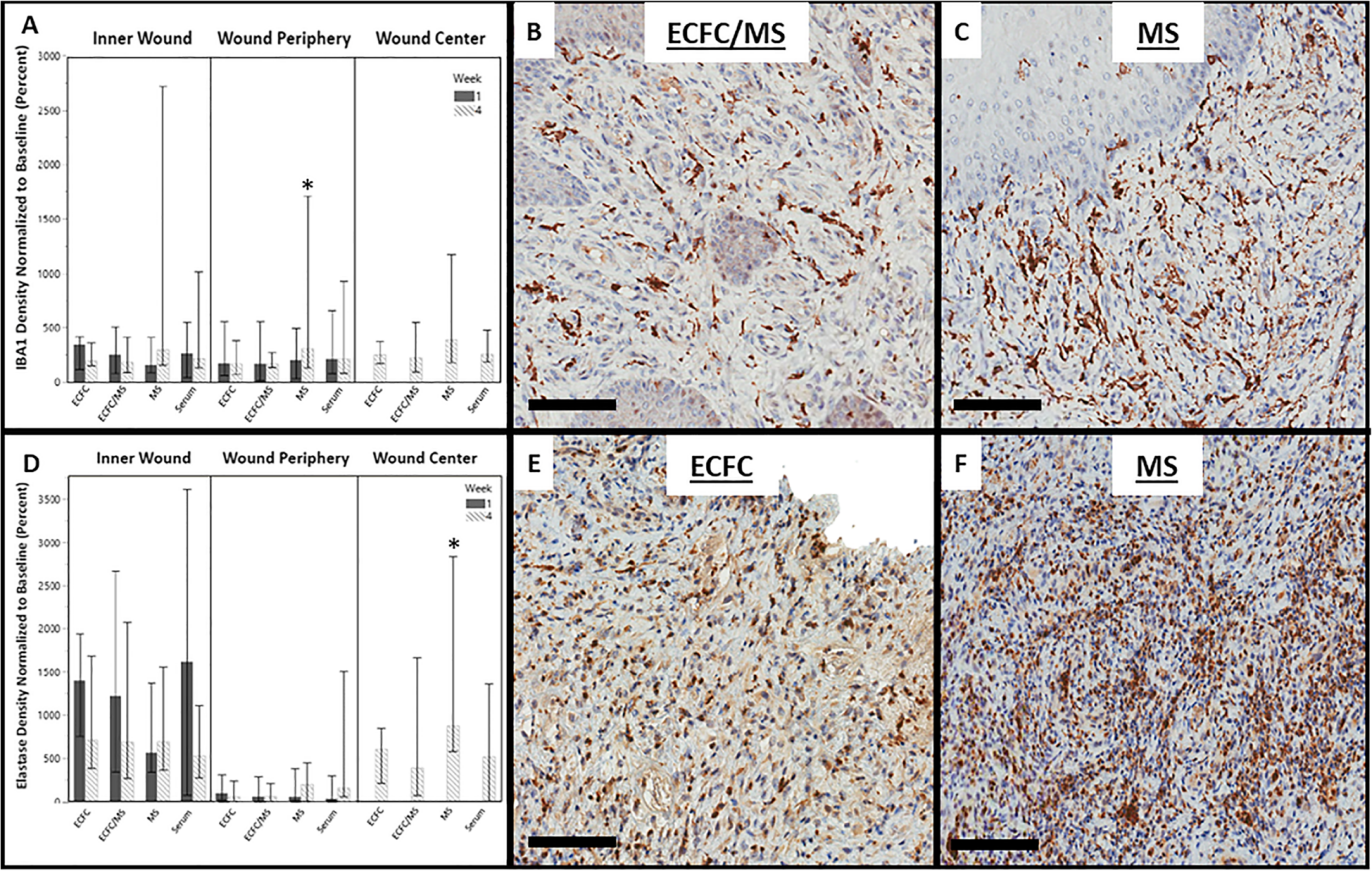
(**a**) Density of IBA1 staining (macrophages) normalized to baseline values at weeks 1 and 4 by treatment group in the Inner Wound, Wound Periphery, and Wound Center regions. Data are presented as median and range. (**b**) Representative photomicrograph of macrophages in tissue at week 4 in the Wound Periphery region of wounds treated with ECFC/MS. (**c**) Representative photomicrograph of macrophages in tissue at week 4 in the Wound Periphery region of wounds treated with MS. (**d**) Density of elastase staining (activated neutrophils) normalized to baseline values at weeks 1 and 4 by treatment group in the Inner Wound, Wound Periphery, and Wound Center regions. (**e**) Representative photomicrograph of neutrophils in tissue at week 4 in the Wound Center region of wounds treated with ECFC (**f**) Representative photomicrograph of neutrophils in tissue at week 4 in the Wound Center region of wounds treated with MS. Note that there is a subjectively greater density of macrophages in the Wound Periphery region of wounds at week 4 treated with MS (**c**) compared to wounds treated with ECFC/MS (**b**); there is also a subjectively greater density of neutrophils in the Wound Center region of wounds at week 4 treated with MS (**f**) compared to wounds treated with ECFCs (**e**). * indicates a significant difference between treatment groups, with MS alone wounds having the highest density of macrophages and neutrophils at week 4 (*P* < 0.05). Scale bars are 100 μm

Comparisons of the inflammatory response (Fig. 6) between treatments were performed on all wounds biopsied at week 1 and week 4 (*n* = 48 wounds from 6 horses, 12 wounds per treatment), and each region of the biopsy (Inner Wound, Wound Periphery, or Wound Center) was analyzed separately. Overall, the acute inflammatory response assessed at week 1 was not different between treatments, but macrophagic and neutrophilic inflammation were decreased in week 4 in ECFC and ECFC/MS treated wounds suggesting ECFC effects on chronic inflammation. The Wound Periphery region in wounds treated with ECFCs and ECFC/MS had significantly less macrophagic (quantified by IBA1 staining) inflammation at week 4 (Fig. 6), with wounds treated with ECFC/MS having significantly less macrophagic inflammation compared to wounds treated with MS alone (*P* = 0.008) There were no differences in macrophage density in the Wound Center and Inner Wound regions (*P* = 0.078 and *P* = 0.064, respectively) at week 4. The effect of treatment group was not significant in the Inner Wound and Wound Periphery regions at week 1 (*P* = 0.232 and *P* = 0.363, respectively).

Activated neutrophil density, as assessed by elastase positive staining, was not different at week 1 but was at week 4 based on treatment group. In the Wound Center regions of wounds at week 4, there were significant differences in neutrophil densities between wounds (*P* = < 0.001) (Fig. 6); the lowest neutrophil densities were found in wounds treated with ECFCs and ECFC/MS. Wounds treated with ECFCs had significantly less neutrophil density as compared to wounds treated with MS alone (*P* = < 0.001). The Wound Periphery regions of wounds at week 4 had no difference in neutrophil density between wounds (*P* = 0.114).

There was not a significant influence of treatment, horse, limb, or week on density of T-cells, as assessed by CD3 positive staining, or B-cells, as assessed by Pax5 positive staining, in this wound model. Densities of T-cells and B-cells were measured and compared to baseline densities. No increase in T-cell or B-cell density was observed compared to baseline for week 1 and week 4 wounds (data not shown).

### Tracking labeled ECFCs

**Fig. 7:**
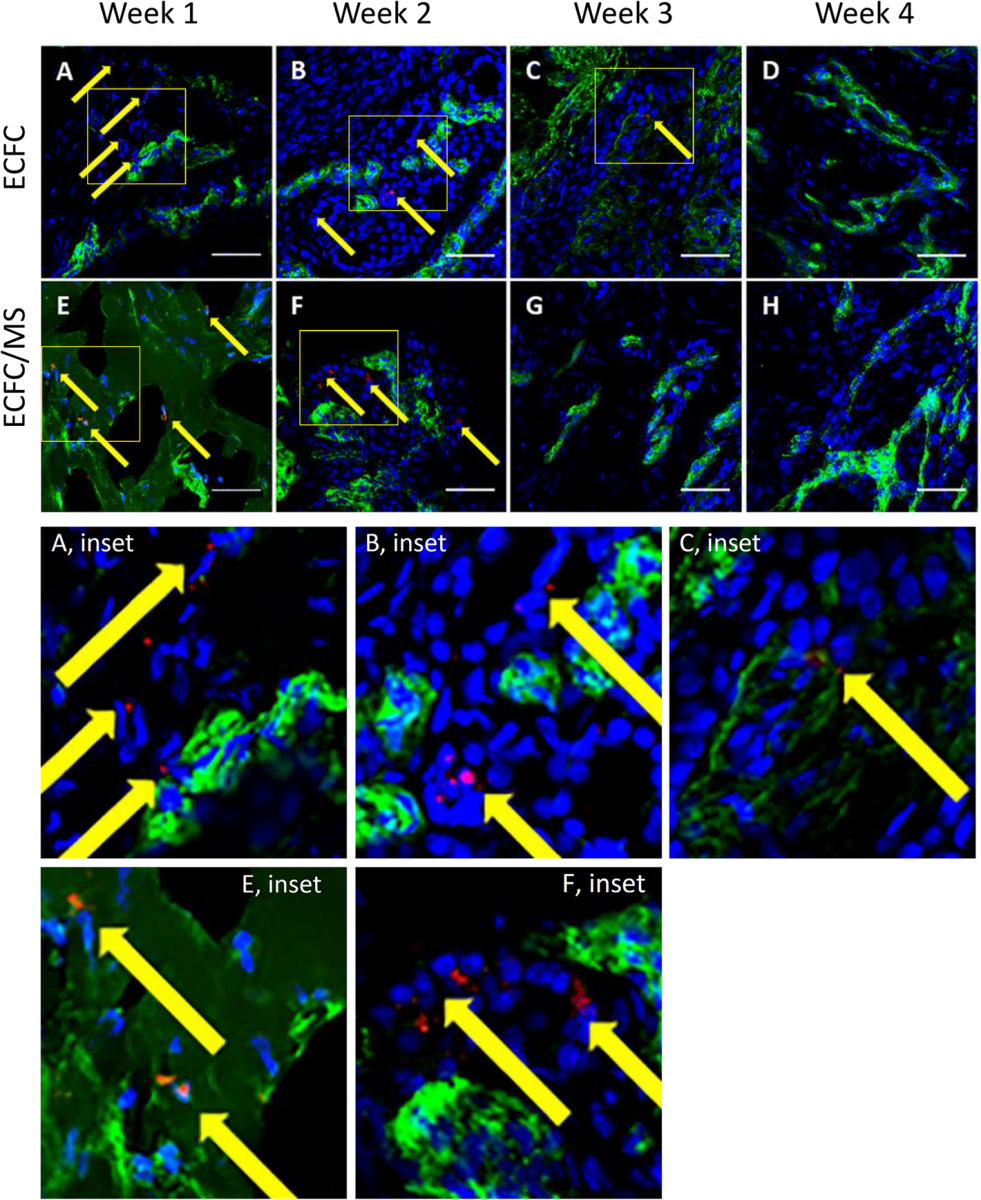
Representative fluorescent photomicrographs of weekly biopsies from wounds treated with ECFCs (**a-d**) and with ECFC/MS (**e-h**) from Phase I horses. The yellow arrows point to QD labeled ECFCs. In (E), PEG-Fb is identified as dark green color with few nuclei. vWF labeled blood vessels are light green. DAPI labeled nuclei are blue. QD are red. Scale bars are 50 μm. Enlarged insets are provided for easier visualization of QD labeled cells

**Fig. 8:**
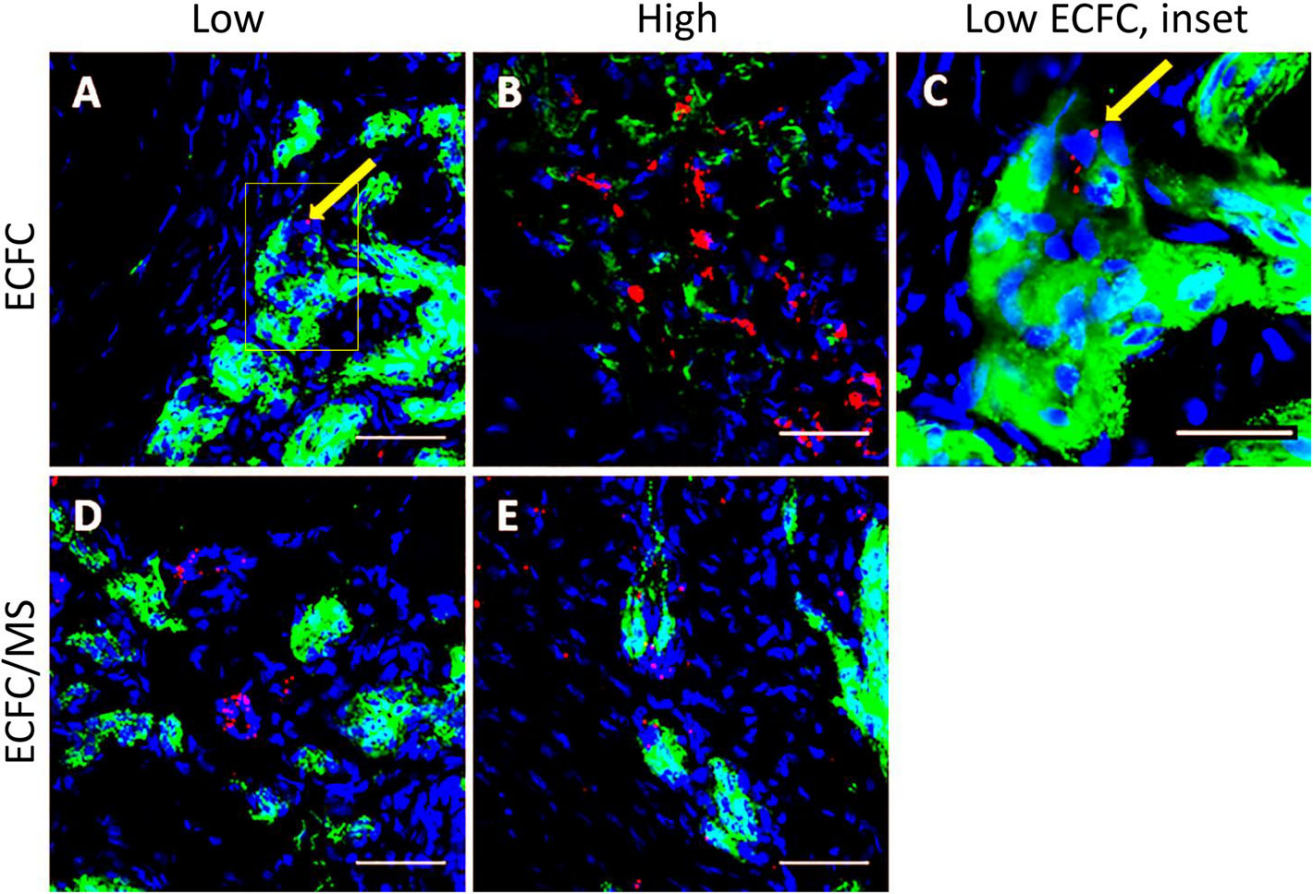
Representative fluorescent photomicrographs of week 1 biopsies from wounds treated with (**a**) 8 million ECFCs per wound (LOW); (**b**) 16 million ECFCs per wound (HIGH); (**c**) Higher magnification of the inset in image A; (**d**) LOW ECFC/MS and (**e**) HIGH ECFC/MS. Note that QD labeled ECFCs are present in all images, and the yellow arrow in (**a**) and (**c**) indicates a QD labeled ECFC incorporated into a blood vessel based on its location within the blood vessel wall. vWF labeled blood vessels are light green. DAPI labeled nuclei are blue. QD are red. Scale bars are 25 μm (**c**) or 50 μm (**a, b, d, e**)

Labeled ECFCs were found up to 3 weeks after injection near blood vessels and also incorporated into blood vessels (Figs. 7,8). In biopsies from horses in Phase 1 with a LOW number of ECFCs and weekly biopsies, labeled ECFCs were found up to three weeks after injection in wounds treated with just ECFCs and up to two weeks after injection in wounds treated with ECFC/MS (Fig. 7). For Phase 2 horses with HIGH number of ECFCs injected that were biopsied at week 1 and week 4, labeled cells were found in wounds treated with both ECFCs and ECFC/MS at week 1 (Fig. 8). At week 4, no labeled ECFCs were observed in any tissue sample for either dosage (LOW vs. HIGH), treatment type (ECFCs vs. ECFC/MS), or injection frequency. The comparisons between the numbers of labeled cells found in week 1 biopsies between different doses of ECFC and ECFC/MS injected wounds are summarized in Table 1. Significantly more labeled cells were identified in ECFC/MS vs. ECFC treated wounds with the LOW dose (Table 1). Labeled ECFCs were commonly found near blood vessels, regardless of whether the wounds were treated with ECFC or ECFC/MS. Rarely, labeled ECFCs could be observed to be incorporated into a newly formed blood vessel (Fig. 8). In wounds treated with ECFC/MS, the PEG-Fb biomaterial was observed in some samples 1 week after injection (Fig. 7). Labeled ECFCs were observed within the PEG-Fb biomaterial in these samples (Fig. 7). The PEG-Fb biomaterial was not observed in any samples after week 1.

**Table 1:** Quantum dot labeled ECFCs in week 1 biopsies

	ECFC	ECFC/MS	*P* value
# Wounds with QD+ cells at week 1	3/6	6/6	0.27
QD+ cells per cryosection ALL (Median [IQR])	11 [3–24]	21 [9–42]	0.14
QD+ cells per cryosection LOW (Median [IQR])	5.5 [3–20.25]	27 [11.75–44.5]	***0.02***
QD+ cells per cryosection HIGH (Median [IQR])	19 [15.75–35.75]	10 [1–21]	0.38

10 slides with 2 sections per slide per biopsy were used to quantify the number of quantum dot (QD) positive ECFCs in week 1 biopsy samples. Each horse had 1 wound per treatment biopsied at week 1. ALL = all cell-treated wounds from week 1 biopsies from both phases of study. LOW = wounds from Phase 1 with 8 mil cells/wound. HIGH = wounds from Phase 2 with 12–16 mil cells/wound. IQR = interquartile range. Significant *P* values are in bold, italicized text.

## Discussion

Our group has demonstrated an effective method of cell delivery using injectable, cell-laden hydrogel microspheres, and we have shown increased vascularization and reduced inflammation in a research model of equine distal limb wounds treated with ECFCs. The effects were modest, but autologous cells from 6 non-athletic horses of various ages and breed were used for this study, and variability of the source cells is an important issue when evaluating clinical efficacy in stem and progenitor cell therapy.

Wounds with EGT are extremely vascular, but microvessels formed within these wounds can be occluded, thus maintaining low wound oxygenation and promoting angiogenesis and fibroproliferation [3]. ECFCs have been studied in small animal models extensively for repair of ischemic tissue. Their vasculogenic effects can be due to direct incorporation of injected ECFCs into damaged blood vessels, formation of de novo blood vessels, or paracrine support [6, 7]. Incorporation of human bone-marrow derived ECFCs into brain capillaries proved beneficial for vascular damage repair in rats with ischemic stroke, and similar benefits after vascular incorporation of injected ECFCs have also been demonstrated in people with intracerebral hemorrhage, acute kidney injuries and myocardial infarctions [7–10]. In a mouse model of hind limb ischemia after femoral artery ligation, labeled ECFCs injected into the tail vein were observed integrated within capillary vessel walls of the ischemic leg by histologic examination 1 to 6 weeks after ligation, and labeled cells were arranged into de novo capillaries within muscle at 6 weeks [6]. ECFCs were also observed in a perivascular location within infarcted myocardium after direct injection, and mRNA analysis revealed that these injected ECFCs upregulated angiogenic and cardiac protective factors [10]. We hypothesized that treatment of wounds with ECFCs would enhance vascularization and reduce inflammation resulting in more rapid wound healing and less EGT formation. Significant effects on inflammation and vascularization were observed despite the large amount of variability in the model; however wound temperature and EGT formation were not different with treatment. The infrared imaging technique utilized in this study can detect changes in temperature due to inflammation and circulation. However, the eschar of some wounds present during healing may have interfered with the emittance of radiant heat, and this may have been a limitation to this imaging modality for this model. Wound size was smaller at week 4 in wounds treated with ECFCs, but these effects were not significant.

Cell therapy is very common in equine practice, but using biomaterials to deliver cells in equine cell therapy is still in its infancy. The use of PEG-Fb in horses has only been reported by our group [21], and this is the first study looking at a clinical effect of a PEG-Fb hydrogel in a horse. This is also the first study evaluating a cellular therapy for wound healing that utilizes PEG-Fb microspheres, a biomaterial cell-delivery vehicle designed in an injectable format. Biomaterial support of cells during delivery is very attractive in order to protect cells during or after injection and increase cell retention after injection [16, 22, 23]. Combining mesenchymal stem cells (MSCs) with an alginate scaffold biomaterial helped to successfully regenerate articular cartilage in a rabbit model [24]. Enhanced early chondrogenesis has been observed when equine MSCs were injected within a fibrin gel biomaterial into an equine model of cartilage defect [25]. Using the PEG-Fb biomaterial to carry therapeutic agents including cells has been investigated in other species and models [21, 23]. Sustained local release of vascular endothelial growth factor and angiopoietin-1 after injection within PEG-Fb microspheres has been demonstrated in a rat myocardial infarction model with intramyocardial injection [23]. We showed that we can consistently create uniform, cell-laden microspheres for in vivo injection, the equine ECFCs survive encapsulation and injection, and PEG-Fb MS encapsulated labeled ECFCs were detected in tissues up to 3 weeks after injection. The PEG-Fb material was observed only up to 1 week after injection, and this is consistent with the ease of hydrogel breakdown in vivo [22, 23]. The fibrinogen backbone of the PEG-Fb biomaterial provides cell adhesion / integrin binding sites found naturally in extracellular matrix proteins, which may have aided cell retention at the point of injection [23, 26]. When we were able to directly visualize the PEG-Fb within tissue sections, we observed no abnormal pattern of vascularization or inflammation associated with this biomaterial. Although the healing rate, vascularization, and number of labeled cells were not different between ECFC treatment and MS encapsulated ECFC treatment, we were able to detect labeled cells in some biopsies from every horse if they were delivered in microspheres; that was not the case for non-encapsulated ECFCs. No clinically significant adverse effects related specifically to cell therapy or microsphere injection were detected in the 6 treated horses. Other than a single fever and mild generalized limb swelling in one horse, no complications from the injections were encountered, and all wounds were completely epithelialized by 2 months.

Equine wound models are frequently used to study wound healing [2, 3, 27–29]. Important differences in wound healing based on anatomic location (body vs. limb, forelimb vs. hindlimb) have been noted in some equine wound model studies [2, 3, 29]. In order to parse out cell effects versus biomaterial effects, multiple control conditions were used in the same horse for our study. Autologous cells were also utilized since injection of allogenic ECFCs has not been evaluated in horses. This led to variation in the model due to biopsies, different limbs, and function of the autologous cells. We did not choose smaller or more numerous wounds just on the forelimbs for this study because of the concern of treatments on one wound affecting another wound that was close by. Future studies may not require as many control conditions.

Based on the relative frequency of ECFCs found near newly formed blood vessels and relative rarity of ECFCs found within the vessel wall, it is possible that ECFCs function in a more paracrine role in neovascularization. The reduced macrophagic and neutrophilic inflammation we observed in cell treated wounds at week 4 could also support a paracrine effect. However further work is required to make this distinction. It is important to note that some vascular tumors such as infantile hemangiomas in humans have been speculated to possibly be caused by endothelial progenitor cells [30]. Although the blood vessel density was greater at week 1 in wounds treated with ECFC or ECFC/MS, this effect was not present at the end of the study, and no excessive vascularization or vascular masses were observed.

In this study, we utilized a histomorphometric software program (Visiopharm, Horsholm, Denmark) to quantitatively evaluate immunohistochemical stain densities in a semi-automated, unbiased manner [31, 32]. This histomorphometric algorithm-based analysis has also been used to quantify degree of angiogenesis in equine large airways [33], however a common approach for wound healing assessment in the equine involves a semi-subjective scoring system [3, 28, 29]. One study of human patients diagnosed with breast cancer found that the histomorphometric software program outperformed traditional pathologist-based analysis of prognostic histologic biomarkers [34]. An automated, algorithm-based approach for immunohistochemical stain analysis is advantageous for quantitative, non-biased results.

Cell labeling and subsequent tracking of injected cells was performed with QD in this study to identify the location of ECFCs at different time points. We have performed in vitro studies with QD in equine ECFCs demonstrating that QD concentrations up to 20 nM do not negatively affect ECFC growth, proliferation, or function [35]. ECFCs labeled with QD were observed up to 3 weeks after injection, and this was independent of whether a low or high number of ECFCs were injected or the number of injections. Given the size of the biopsy samples, it is also possible that the QD labeled ECFCs were near and in the wound, but they were not in the specific slices of tissue analyzed.

## Conclusions

This study highlights that equine ECFCs with or without encapsulation in PEG-Fb microspheres can be administered locally to distal limb wounds without adverse effect. This is the first report of clinical effects of ECFCs and of PEG-Fb hydrogel microspheres in the horse. Wounds that were treated with ECFCs with or without PEG-Fb encapsulation had increased vascularization acutely and decreased neutrophilic and macrophagic inflammation chronically, which may be an advantageous to wound healing. No clinically relevant changes were observed in GS or wound size due to treatment, but the observations and cell tracking pave the way for future studies using ECFCs or other cell types combined with the injectable PEG-Fb microspheres.

## Methods

### Horses

All procedures involving animals were approved by the Auburn University Institutional Animal Care and Use Committee, protocol #2014–2637. Six, healthy university-owned, adult horses were utilized for autologous ECFC isolation and to create the distal limb wound model. Breeds included warmblood (*N* = 2), quarter horse (*N* = 1), Tennessee walking horse (*N* = 1), thoroughbred (*N* = 1), and a thoroughbred cross (*N* = 1). They underwent general physical examinations before recruitment into the study. Horses were kept free in individual box stalls in an environmentally-controlled building for the duration of the study (4 weeks) and allowed ad libitum access to grass hay and water. Horses were examined daily for signs of discomfort, lameness, and systemic illness. At the termination of the study, the animals were monitored until all wounds were completely healed, and then returned to their herds.

### Autologous ECFC isolation and labeling

Whole blood was collected from either the cephalic vein or the jugular vein for autologous ECFC isolation using either a whole blood isolation or density gradient centrifugation isolation method as previously described [36, 37]. Briefly, blood samples taken from the jugular and cephalic veins were mixed with endothelial-specific cell culture medium and placed in collagen-coated cell culture flasks without additional processing (whole blood method) or after isolation of the monocellular plasma layer (density gradient centrifugation method). Both methods and collection sites were used in order to maximize chances of isolating autologous ECFCs. ECFCs were characterized based on cell surface markers and in vitro formation of tubules on basement membrane and uptake of low density lipoprotein [36, 37]. ECFCs were cultured in 75 cm^2^ cell culture flasks with endothelial cell growth medium (EGM-2) at standard cell culture conditions (37 °C, 5% CO_2_, 95% humidity) with the manufacturer-supplied growth factors and anti-microbials (Lonza, Visp, Switzerland) and 10% equine serum (HyClone Laboratories Inc., Logan, Utah). ECFCs of passage 3–5 were labeled with 4 nM semiconductor QD (Invitrogen, CA, USA) for in vivo cell tracking [35].

### Encapsulation of ECFCs

PEG-fibrinogen was synthesized using a previously published method [26]. Briefly, fibrinogen was dissolved and then slowly added to PEG-diacrylate to react for 3 h in the dark at room temperature. The PEGylated fibrinogen was extracted with acetone, centrifuged to remove the acetone, and dialyzed in sterile phosphate buffered saline (PBS) for 24 h at 4 °C without light. Cell encapsulation in the PEG-fibrinogen hydrogel was achieved using a custom microfluidic polydimethylsiloxane (PDMS) system [21]. The microfluidic PDMS mold was sonicated in 70% ethanol for sterilization.

PEG-Fb precursor solution was prepared with 0.1% (w/v) of Pluronic F68 (Sigma, St. Louis, MO), 0.1 mM of EosinY photoinitiator (Fisher Scientific, Waltham, MA), 1.5% (v/v) triethanolamine (Acros Organics, Waltham, MA), and 0.39% (v/v) of N-vinylpyrrolidone (Sigma, St. Louis, MO). ECFCs were detached from the tissue culture flask, centrifuged, and then resuspended in the PEG-Fb precursor solution at a cell density of 10 million cells/mL. The suspension of cells in polymer precursor solution was infused through the top inlet of the microfluidic device, and mineral oil was infused through the bottom inlet. MS were formed by emulsification at the junction with the outlet channel and crosslinked using a 2.8 W/cm^2^ full spectrum visible light (Prior Scientific, Rockland, MA). The cell-laden PEG-Fb MS (ECFC/MS) were washed twice with medium to remove the residual mineral oil and cultured in EGM-2 at 37 °C and 5% CO_2_. Cell viability after encapsulation was tracked in subset of ECFC/MS using an XTT cell viability assay (Biotium, Fremont, CA).

### Creation of distal limb wounds

On day 0, horses were restrained in stocks and sedated with detomidine hydrocholoride (0.01 mg/kg; IV) and butorphanol tartrate (0.04 mg/kg; IV). The dorsolateral surfaces of both metacarpi and metatarsi were clipped and aseptically prepared. Perineural anesthesia of the median cutaneous antebrachial nerve, deep and superficial peroneal nerves and ring blocks was performed using 2% mepivacaine hydrochloride. Two, full thickness, 2.5 cm X 2.5 cm (6.25 cm^2^) wounds were created on each metacarpus and metatarsus using a sterile wound template and a #15 scalpel blade; this skin was kept as the baseline sample for immunohistochemistry. Each horse had 2 wounds created per limb (fore and hind) for a total of 8 wounds per horse (Fig. 8). The wounds were bandaged for approximately 24 h for hemostasis and healed by second intention for the duration of the 4-week study.

### Experimental phases

Wounds were injected with 4 different treatments with two phases of the study (Fig. 1). Treatments included equine serum (HyClone, Logan, UT), ECFCs suspension in serum, acellular PEG-Fb MS suspended in serum, and ECFCs-laden PEG-Fb MS (ECFC/MS) suspended in serum. Each wound was randomly assigned to one of the 4 treatments using a random number generator, resulting in 2 wounds per treatment per horse. All investigators involved in wound assessment or daily care of the horses were blinded to the treatment assignments.

In Phase 1, three horses had treatments injected around the wound periphery at a single time point (24 h after wound creation, day 1). ECFC and ECFC/MS treated wounds were injected at the mid-point of each wound edge with 2 million cells for a total of 8 million cells/wound. For the 2 wounds with the same treatment, one wound was biopsied weekly, and the other wound was only biopsied at the end of the study. Phase 1 horses had the following biopsy schedule: 4 wounds (1 from each treatment) were biopsied at the medial leading edge and at the center of the wound at week 4. The other 4 wounds (one from each treatment) were biopsied at the leading edge each week (order of biopsy location was: medial, lateral, ventral, then dorsal) for 4 consecutive weeks and from the center of the wound at week 4.

In Phase 2, three horses had treatments injected into 4 wounds at a single time point (24 h after wound creation, the same as described in phase 1) and into 4 wounds at 2 time points (24 h and 1 week post wound creation). Four wounds (1 per treatment) were injected once with 8 million cells/wound (LOW) for ECFC and ECFC/MS groups (day 1), and the medial leading edge and the center of the wound were biopsied at 4 weeks. The other 4 wounds (one per treatment) were injected twice with a higher dosage of cells: once at 24 h with 16 million cells/wound (4 injections on 4 edges with 4 million cells/injection), and then again at week 1 with an additional 12 million cells/wound (3 injections on 3 edges with 4 million cells/injection and 1 biopsy on 1 edge) (HIGH). For the 4 wounds with 2 treatment injections, biopsies were collected from the medial leading edge of the wound at week 1 (before the second injection) and then from the lateral leading edge and the wound center at 4 weeks.

Wounds were analyzed non-invasively each week of the study, and biopsies were analyzed by IHC for vascular formation and inflammation and by indirect immunofluorescence (IF) for cell tracking.

### Wound treatments

To perform injections of treatments for each wound, horses were sedated and perineural anesthesia was administered as described above. The treatments were injected subcutaneously at the mid-point of each edge of the wound using 18 gauge × 1″ needles on 1 mL syringes with a volume of 600 (LOW) – 1000 (HIGH) μL. Syringes contained either either 2 million cells (LOW) or 4 million cells (HIGH) with approximately 2800 ECFCs per MS (LOW treatments had 750 MS, HIGH treatments had 1500 MS).

### Wound biopsies

For the surgical punch biopsy procedure, horses were sedated and local anesthesia administered as previously described. Wounds were cleaned with water and sterile 0.9% NaCl. Depending on timepoint, biopsies (2 per site) were obtained at the leading edge of the wound with the punch instrument overlapping the visual epithelization or at the center of the wound in the granulation bed. One biopsy sample was formalin fixed and paraffin embedded, and the other was embedded in optimal cutting temperature compound and snap frozen in liquid nitrogen cooled isopentane.

### Non-invasive wound assessment

The WSA was measured weekly with digital photography. Each wound was photographed at a 40 cm distance with a metric ruler immediately adjacent to the wound for scale, and the area of the unhealed wound at each time point was calculated using Image J software (imagej.nih.gov/ij/). WSA was calculated as percentage of the original wound size on day 0 for non-biopsied wounds. Granulation tissue formation was scored weekly by two, blinded investigators (AAW, FJC). Each observer scored all wounds independently based on 3 clinically-relevant parameters: protrusion of granulation tissue (0 = none; 1 = mild; 2 = marked); character of granulation tissue (0 = smooth; 1 = rough); and color of granulation tissue (0 = pink; 1 = yellow or dark red) [38]. Protuberance was weighted 50%, and quality and color of the granulation tissue was weighted 25%. The observers’ scores were averaged, with the total weighted GS maximum of 1.5 for each wound.

Prior to the study, 24 h after wound creation, and each week for the duration of the study, thermographic images were obtained with a hand-held infrared imaging radiometer placed at a distance of 45 cm from the distal limb, perpendicular to the wound surface (FLIR B360, Optics Planet, Northbrook, IL). Infrared imaging emissivity was set at 0.98. Horses were acclimated to the temperature controlled imaging area for 15 min prior to image aquisition. Temperature readings were obtained from each edge of the wound (medial, lateral, proximal, distal), the wound center, and 1 cm above and 1 cm below the wound. Edge measurements (medial, lateral, proximal, distal) were averaged for analysis as one value. Mean temperature for each anatomic area was calculated using the recorded ambient temperature and humidity to ensure accurate measurements. The percent change in temperature at all time points from baseline (24 h after initial surgery) was used for analysis. The entirety of the study was during summer months to ensure no cold-temperature related distal limb vasoconstriction.

### Tissue staining and immunohistochemistry

Tissue sections (4 μm) were stained with hematoxylin and eosin to evaluate structure and with Masson’s trichrome (Polysciences, Warrington, PA) to quantify collagen formation. The IHC staining was performed on formalin fixed paraffin embedded tissue with the following: elastase to quantify activated neutrophil density [39, 40]; ionized calcium-binding adapter molecule 1 (IBA1) to quantify macrophage density [41]; vWF to quantify vascularization [42]; CD3 to quantify T-cell density [43]; and paired box 5 (Pax5) to quantify B-cell density [44]. Immunohistochemistry was performed at room temperature with primary antibodies diluted in 3% PBS as follows: polyclonal rabbit anti-human neutrophil elastase antibody diluted at 1:1000 for 30 min (Abcam 68,672, Cambridge, MA); polyclonal rabbit anti-mouse IBA1 diluted 1:750 for 30 min (CP290, Biocare Medical, Pacheco, CA); polyclonal rabbit anti-human vWF manufacturer-diluted for 15 min (A0082, Dako, Santa Clara, CA); polyclonal rabbit anti-human CD3 manufacturer-diluted for 15 min (GA503, Dako, Santa Clara, CA); and monoclonal mouse anti-human Pax5 manufacturer-diluted for 20 min (M7307, Dako, Santa Clara, CA). Immunodetection was achieved with a horse radish peroxidase enzyme labeled polymer (Dako, Santa Clara, CA) followed by conjugated rabbit secondary antibody, 3,3′-diaminobenzidine solution, and counterstaining using hematoxylin.

Stained slides were digitally scanned at a high resolution (Aperio, Leica Biosystems, Buffalo Grove, IL), and the stains quantified using a commercially available software program designed for histologic image analysis (Visiopharm®, Horsholm, Denmark). Specific regions were designated “Inner Wound” (non-healed wound within advancing line of epithelialization), “Wound Periphery” (skin with epithelial cell cover, just on the periphery of the advancing line of epithelialization), and “Wound Center” (area at the center of the non-healed wound); the staining in these regions was quantified separately. Inner Wound and Wound Periphery regions were found in biopsies from the leading edge (analyzed at weeks 1 and 4), and the Wound Center region was from the biopsy taken from the center of the wound only at week 4. The software program quantified positively staining pixels / area and expressed as density: positive stain quantity / (positive stain quantity + non-stained quantity). Density values were then normalized to the individual baseline values for that wound as: [(density value – baseline density value) / baseline density value] × 100.

### Indirect immunofluorescent staining and confocal microscopy

Frozen tissue sections (20 μm thickness, 10 slides per wound with 2 sections per slide) were fixed in 4% paraformaldehyde, permeabilized with 0.3% Triton, and incubated with a polyclonal rabbit anti-human vWF antibody (1:200, A0082, Dako, Santa Clara, CA). Detection was performed using a goat anti-rabbit Alexa Fluor 488 secondary (1:400, A11008, Life Technologies, Grand Island, NY) and 4′, 6-diamidino-2-phenylindole (DAPI) solution. Sections were imaged using fluorescent and confocal microscopy to detect vWF positive cells and QD labeling. The number of QD-labeled ECFCs was totaled for each cryosection.

### Statistical analysis

Data were analyzed using commercially available software (JMP Pro 13.0 and SAS 9.4). Descriptive data were analyzed for normality using a Shapiro Wilk test or Q-Q plot and expressed as mean +/− SD or median (range) as appropriate. Thermographic data, WSA, granulation tissue scoring, and IHC stain quantification were assessed with a General Linear Model or a mixed model with repeated measures to assess the effect of individual horse as a random factor, and treatment groups, time point, wound location (forelimb vs hind limb), and effect of biopsy as fixed factors. Analysis of WSA was performed using a mixed model with repeated measures on wounds that were only biopsied at 4 weeks, so the statistical model for this variable assessed the effects of time point, wound location, and treatment. Analyses for thermographic data and granulation tissue scoring utilized a generalized linear model that included data from all wounds on all horses, so the effects of the time point, wound location, biopsy, and treatment (including ECFC injection dose and frequency) were included in the statistical model. Quantification of IHC stains was only performed on wounds biopsied once at either week 1 or week 4, so the statistical model for IHC stain quantification was a generalized linear model that included treatment and wound location and not time point since the same wound was not biopsied at each time point. Cell tracking descriptive data was obtained by week, and the number of QD-labeled cells was analyzed for influence of presence or absence of PEG-Fb MS encapsulation and dose of ECFCs injected. Tukey-Kramer tests were used to analyze differences between levels. Pearson correlation coefficients were used for associations between continuous variables. Associations between categorical variables were analyzed with a Fischer’s exact test. *P* < 0.05 was considered significant.

## Data Availability

The datasets analyzed during the current study are available from the corresponding author on reasonable request.

## Abbreviations

CO_2_: Carbon dioxide
DAPI: 4′, 6-diamidino-2-phenylindole;
DMSO: Dimethyl sulfoxide
ECFC/MS: Endothelial colony forming cells encapsulated into poly(ethylene) glycol-fibrinogen microspheres
ECFCs: Endothelial colony forming cells
EGM-2: Endothelial growth medium
EGT: Exuberant granulation tissue
EPCs: Endothelial progenitor cells
Fb: Fibrinogen
FBS: Fetal bovine serum
GS: Granulation score
IBA1: Ionized calcium-binding adapter molecule 1
IF: Immunofluorescence
IHC: Immunohistochemistry
MS: Microspheres
MSCs: Mesenchymal stem cells
OR: Odds ratio
Pax5: Paired box 5
PBS: Phosphate buffered saline
PDMS: Polydimethylsiloxane
PEG: Poly(ethylene) glycol
PEG-Fb: Poly(ethylene) glycol coupled with fibrinogen
QD: Quantum dots
vWF: Von Willebrand factor
WSA: Wound surface area
